# Influence of Different Treatments on the Structure and Conversion of Silicon Species in Rice Straw to Tetraethyl Orthosilicate (TEOS)

**DOI:** 10.1002/open.202300111

**Published:** 2023-08-07

**Authors:** Qianxin Sun, Shanshan Feng, Guiying Li, Yue Qi, Changwei Hu

**Affiliations:** ^1^ Ministry of Education College of Chemistry Sichuan University Chengdu Sichuan 610064 P. R. China

**Keywords:** biomass, green chemistry, renewable resource, silicon, tetraethyl orthosilicate

## Abstract

The production of tetraethyl orthosilicate (TEOS) from biomass provides a new way for TEOS production and biomass valorization. In this study, rice straw was treated using different fractionation methods, and the content, state, and reactivity of Si in the treated samples were investigated. It was found that acid treatment and ethanol extraction kept most Si in the biomass, while alkali treatment caused significant Si loss. Si was mainly present in the SiO_x_, Si−O−C, and Si−O−Si states in the surface of raw rice straw, cellulose and Klason lignin. The results showed that the Si−O−Si state in rice straw was beneficial for the formation of TEOS. The removal of lipids from rice straw facilitated the production of TEOS, giving the highest TEOS yield of 76.2 %. In contrast, the production of TEOS from other samples became difficult; the simultaneous conversion of the three organic components of rice straw also facilitated the production of TEOS.

## Graphical abstract

The effect of different treatment methods on Si content and state as well as factors influencing the generation of tetraethyl orthosilicate, TEOS, have been investigated. The results showed that the removal of lipids from rice straw facilitated the production of TEOS, giving the highest TEOS yield of 76.2 %, and the Si−O−Si state in rice straw was beneficial for the formation of TEOS.

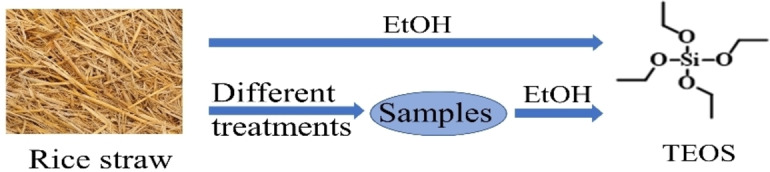

## Introduction

Human survival and development depend on non‐renewable fossil resources. However, the scarcity of fossil resources has attracted people‘s attention in recent years. Biomass is a type of abundant renewable resource, the valorization of which has attracted much attention. Nearly half of the world‘s population lives on rice,^[1]^ the largest cereal crop on earth. According to the statistics released by the US Department of Agriculture in 2022, current global annual rice production is about 500 Mt. Moreover, a large amount of rice straw residue, having a volume of 45 % of rice, is produced with rice production, which is the richest and most renewable resource available. However, nearly half of the straw is burned resulting in a huge impact on the environment.[^2^, ^3^] Rice is a typical Si‐accumulating plant, and the Si content in the shoot can be up to about 10 %.[^4^, ^5^] Thus, rice straw is composed of cellulose, hemicellulose, lignin, and a significant amount of Si, and the biogenic silicon has attracted considerable interest. For example, silica from rice husk ash can be reduced using a carbon source such as wood, charcoal, or coal at temperatures of ≥1700 °C or higher to produce silicon, or reduced to SiC, Si_3_N_4_, or Si_2_N_2_O.[^6^, ^7^] Another way is to pre‐treat the rice husk ash with acid to remove other trace metals, and then roast it to produce silica with different pore sizes.[^8^, ^9^] However, the above applications of bio‐silica still involve combustion during the preparation of ash, which can have detrimental effects on the ecosystem.

In our previous work, using waste enzymatic lignin as raw material and ethanol as solvent, alkoxysilanes were found in the reaction products.^[10]^ Alkoxysilanes especially tetraethyl orthosilicate (TEOS) have many applications. It shows excellent performance in many fields, such as the photovoltaic industry, biomedicine, information technology, and construction.[^11^, ^12^, ^13^, ^14^, ^15^, ^16^] For example, it is used as a silicon source for organic synthesis, for the synthesis of various SiO_2_ (catalyst supports, etc.) with controlled particle size, morphology, and porosity, for the preparation of optical glasses, coatings, adhesives, modifiers, etc. However, the current production of TEOS in industry usually uses SiCl_4_ as starting material. On one hand, the process is energy‐intensive, on the other hand, it is harmful to the environment and ecology because of the use and production of large amounts of HCl, although it could be recycled. In recent years, some researchers have invented new ways to generate TEOS. For example, R. Laine et al.^[17]^ used SiO_2_ as starting material to react with excess ethylene glycol under the catalysis of a strong base to produce spirosilane firstly, which was then subjected to alcohol exchange to get TEOS. Similarly, N. Fukay et al.[^18^, ^19^] used strong bases and a significant amount of dehydrating agent to promote the reaction of SiO_2_ with ethanol to get TEOS. These studies have greatly enriched and advanced the methods for the preparation of alkoxysilanes. However, current studies using SiO_2_ as starting material usually require multi‐step reactions and the use of strong bases (Table S1). Therefore, there is an urgent need to develop new, efficient methods for the synthesis of alkoxysilanes, and it is of great significance to study the generation of TEOS from biomass.

Si co‐exists with different components of biomass. Some studies have shown that silica deposited in specialized cells or on cell walls.[^20^, ^21^] In Si‐cell wall complexes, possible Si ligands include those from hemicellulose, pectin, and lignin, besides, Si may bond with hemicellulose in the state of Si−O−C.[^22^, ^23^, ^24^, ^25^] Because of the difference in the interactions between Si and the three main organic components of biomass, each component may have a different effect on the production of TEOS in biomass. However, the formation of TEOS from Si in different components and different states of Si is unclear. Based on these premises, in this work, we prepared lipids‐free material, lignin‐rich material, holocellulose‐rich materials, and cellulose‐rich materials from rice straw. The content and state of Si in different samples were studied. Meanwhile, the formation of TEOS from the seven samples and the factors influencing the formation of TEOS were also analyzed to better understand the formation of TEOS from biomass.

## Results and Discussion

### The amount of Si remained in the samples from differently treated rice straw

The Si content in raw rice straw (RS) and differently treated materials were shown in Table 1. There are 6.6 % Si in RS. After lipids extraction, the Si content in lipids‐free rice straw (LFRS) increased to 7.2 %, although 4.3 % Si in the raw material was lost caused by the removal of lipids. For National Standard (GB) method prepared holocellulose (HCGB), 19.6 % silicon was also removed with the removal of lignin. With further removal of hemicellulose, more silicon was lost because of the use of alkali. For the alkali method‐prepared holocellulose (HCAK) and alkali method‐prepared alpha‐cellulose (ACAK), the use of NaOH/H_2_O_2_ resulted in most of the silicon being dissolved, and the Si content in HCAK and ACAK was only 0.9 % and 0.1 % respectively. For Klason‐lignin (KL), prepared under acidic conditions using sulfuric acid, most of the silicon was kept in KL. In total, in the fractionation process of rice straw, Si has different degrees of loss. Acid treatment and ethanol extraction resulted in less Si loss, while alkali treatment caused significant Si loss.

**Table 1:** The content of the components of differently treated rice straw samples.

Samples	Lignin^[a]^ (%)	Cellulose^[a]^ (%)	Hemicellulose^[a]^ (%)	Ash^[a]^ (wt.%)	SiO_2_^[b]^ (wt %)	Si^[c]^ (%)
RS	10.1	33.8	15.3	10.7	6.6	100
LFRS	11.5	38.4	17.4	11.7	7.2	95.7
HCGB	4.4	43.7	18.7	10.7	10.4	80.4
ACGB	3.7	85.3	8.4	8.8	8.7	37.7
HCAK	0.3	79.9	12.4	1.0	0.9	4.5
ACAK	1.4	98.9	3.7	0.4	0.1	0.4
KL	64.3	–	–	35.7	35.7	95.7

[a] The content of lignin, cellulose, hemicellulose, and ash were measured by NREL methods. [b] The content of SiO_2_ in the sample. [c] Related to the total Si content in raw rice straw. RS, raw rice straw; LFRS, lipids‐free rice straw; HCGB, GB method prepared holocellulose; ACGB, GB method prepared alpha‐cellulose; HCAK, alkali method prepared holocellulose; ACAK, alkali method prepared alpha‐cellulose; KL, Klason‐lignin.

### FT‐IR Analysis

**Figure 1:**
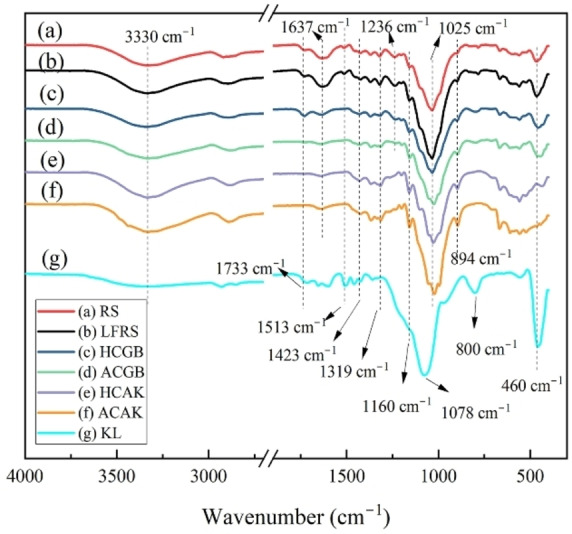
FT‐IR spectrum of raw materials (a) RS, (b) LFRS, (c) HCGB, (d)ACGB, (e) HCAK, (f) ACAK, and (g) KL. RS, raw rice straw; LFRS, lipids‐free rice straw; HCGB, GB method prepared holocellulose; ACGB, GB method prepared alpha‐cellulose; HCAK, alkali method prepared holocellulose; ACAK, alkali method prepared alpha‐cellulose; KL, Klason‐lignin.

The IR spectra of the materials were shown in Figure 1. The broad absorption band near 3330 cm^−1^ was observed in all spectra, which could be attributed to the ‐OH groups of the structure of cellulose, hemicellulose, and lignin. The bands at 2925 cm^−1^ and 1423 cm^−1^ were ascribable to ν(C−H) and δ(C−H), which indicated the existence of methyl and methylene groups, and the peak at 1319cm^−1^ was attributed to C−H bending. For RS, LFRS and KL, the spectrum at ~1733 cm^−1^ was attributed to the acetyl groups or ester bonds of hemicellulose, or the ester bonds in the carboxyl groups of ferulic and *p*‐coumaric acid of lignin.[^26^, ^27^] This peak disappeared in ACAK, HCAK, and GB method‐prepared alpha‐cellulose (ACGB), which indicated the successful isolation of lignin and hemicellulose. In HCGB, the band at ~1733 cm^−1^ illustrated the remaining of hemicellulose and lignin, which was also confirmed by component determination of the materials (Table 1). Moreover, the peaks noticed at 1637 cm^−1^ and 1236 cm^−1^ denote C=C and C−O stretching of acetyl groups in the lignin component in the spectra of Figure 1 (a, b, and g).^[27]^ The aromatic skeletal vibration signal was observed at 1513 cm^−1^ and this band did not exist in cellulose and holocellulose caused by the removal of lignin.[^28^, ^29^]

The strongest and sharp peak at around 1025cm^−1^ in the FTIR spectra of plant fiber indicated the high cellulose content in samples (Figure 1a–f). Meanwhile, the existence of the weak peak at 1160cm^−1^ was assigned to C−O−C stretching vibration, and it was attributed to the glycosidic linkage in cellulose.^[30]^

Because of the removal of cellulose and hemicellulose, glycosidic linkage was not observed in Figure 1g. Furthermore, the weak band at 894cm^−1^ was the characteristic of the β‐glycosidic bonds among the cellulose units, and this band was also observed in previous literature.[^26^, ^27^, ^31^] With the removal of lignin and hemicellulose, the bond at 894 cm^−1^ became more pronounced, in agreement with the result of cellulose content (Table 1). The strong peaks at 1078 cm^−1^, 800 cm^−1^, and 460 cm^−1^ in KL (Figure 1g) were attributed to the high SiO_2_ content. The band around 1080 cm^−1^ and 800 cm^−1^ was attributed to the Si−O−Si asymmetric and symmetric stretching of silica. The bands around 460 cm^−1^ could be attributed to the stretching vibration of the Si−O bonds.[^32^, ^33^, ^34^] The bands around 800 cm^−1^ and 460 cm^−1^ were also observed in RS, LFRS, HCGB and ACGB. While, in HCAK and ACAK (Figure 1e and 1 f), these bands were not observed, which may be attributed to the removal of SiO_2_ during the treatment with strong base. This was consistent with the results of Si content (Table 1).

### The surface state of Si in differently treated samples.

**Figure 2:**
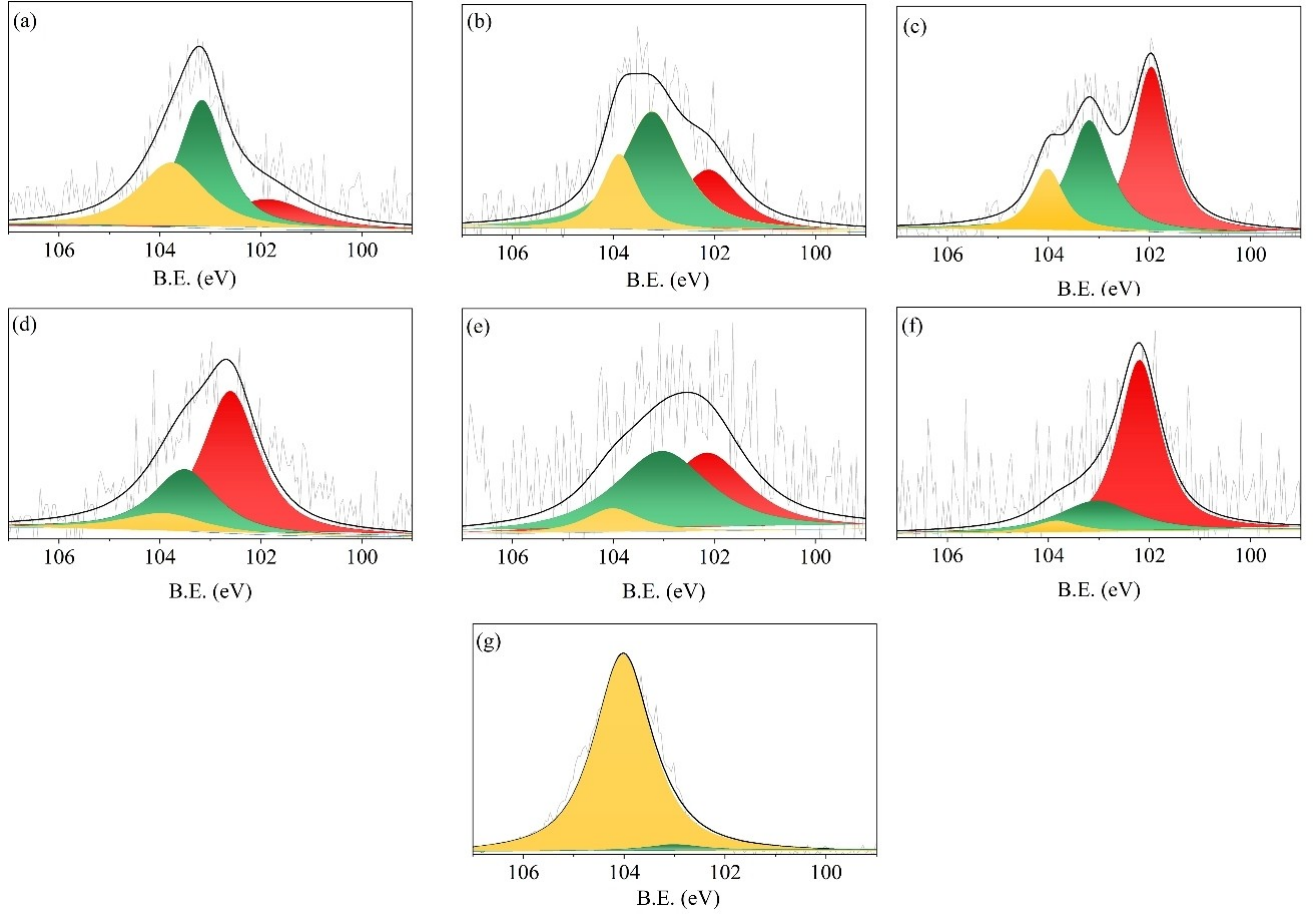
The typical XPS spectra of Si 2p: (a) RS, (b) LFRS, (c) HCGB, (d) ACGB, (e) HCAK, (f) ACAK, and (g) KL.

The XPS spectra of Si 2p were shown in Figure 2. For differently treated samples, a broad distribution at 100.5–104.5 eV indicated the existence of silica (SiO_x_ at 104.0 eV), silicate/(poly)silicic acid (Si−O−Si at 103.3 eV), and Si−O−C (102.2 eV) species.[^35^, ^36^] For RS and LFRS, surface silicon was mainly in the state of Si−O−Si. With the removal of lignin, the Si−O−Si species decreased, while the Si−O−C species significantly increased. The Si−O−Si and Si−O−C were the main surface states of Si in HCGB and HCAK (Figure 2c and 2e). In ACGB and ACAK (Figure 2d and 2 f), Si−O−Si species decreased further. Alkali would cause the silicon to be removed, and the percentage of Si−O−Si species decreased. However, for ACAK, although strong alkali was used in the preparation process, it still contained 0.1 % Si, and the main state of Si was Si−O−C. This meant that the Si−O−C state was very stable and had a very low reactivity.^[37]^ For KL (Figure 2g), the small peak centered at 103.3 eV corresponded to Si−O−Si species, and the predominant peak at 104.0 eV was SiO_x_ species, while Si−O−C species were destroyed with the decomposition of polysaccharides by using the NREL method. Although 95 % of Si in RS was kept, the state of Si changed in the preparation of KL. This implied that the state of Si cannot be fully retained as that in RS after treatment.

### SEM Analysis

**Figure 3:**
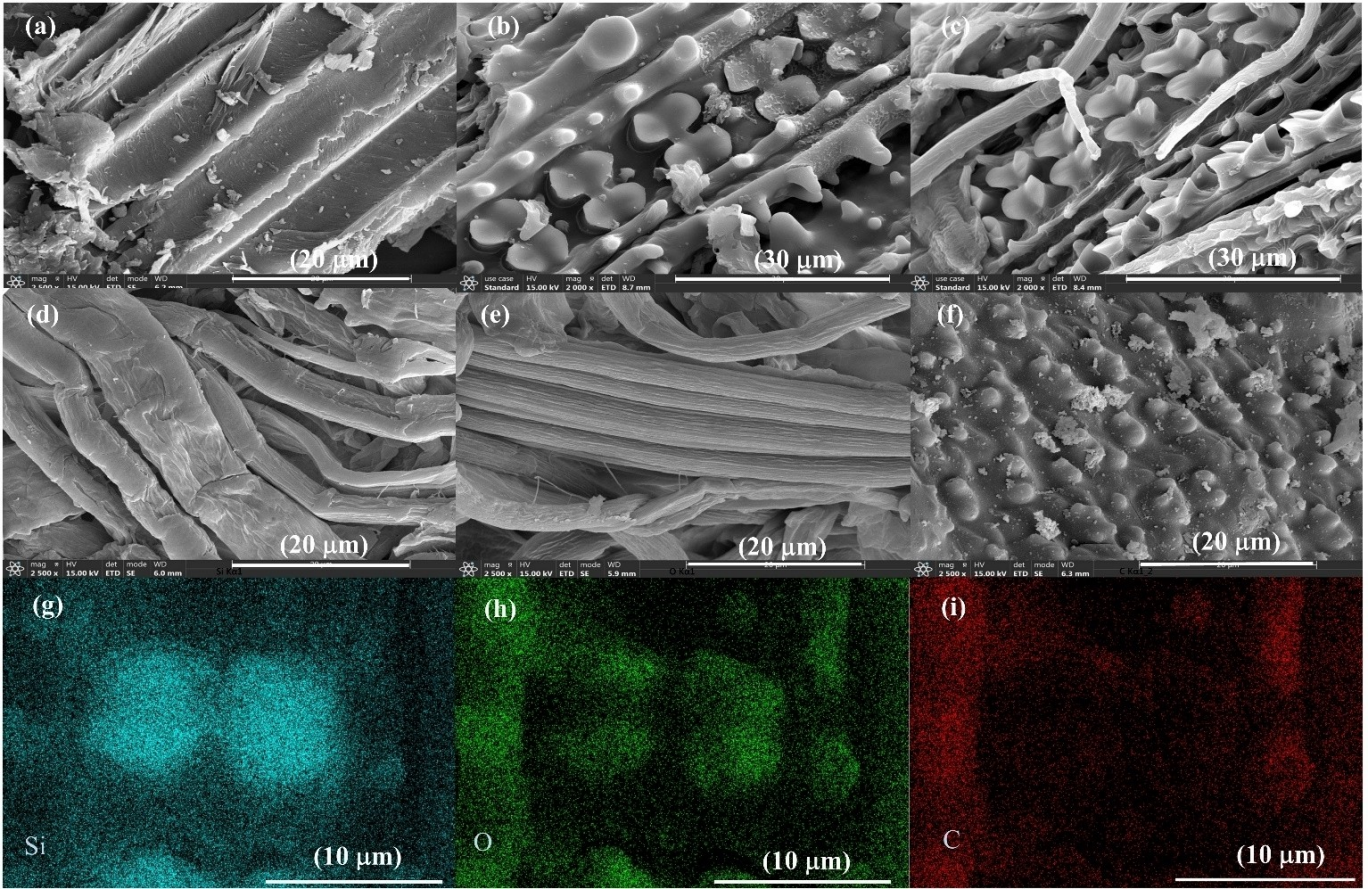
SEM micrographs of materials: (a) LFRS, (b) HCGB, (c) ACGB, (d) HCAK, (e) ACAK (f) KL and mapping images of dumbbell‐shaped silica body from HCGB, (g) Si, (h) O, (i) C.

The SEM images of the materials were shown in Figure 3. During the growth of rice straw, silicic acid (H_4_SiO_4_) was absorbed from the soil solution, and silica cells in the leaf epidermis developed gradually into a dumbbell shape and became increasingly silicified as leaves aged.^[38]^ In this study, after lignin removal, the dumbbell‐shaped silica body was observed in HCGB and ACGB prepared using the GB method. However, the silica body was not found in HCAK and ACAK prepared by the alkali treatment method. This indicated that the method of GB had little effect on the dumbbell‐shaped silica body, and the method of AK destroyed the dumbbell‐shaped silica body in the preparation of holocellulose and alpha‐cellulose. The SEM mapping images of the dumbbell‐shaped silica body from HCGB were magnified and the elemental distribution was observed. Red, green, and blue colors were used to present the elements of C, O, and Si. As a result, both Si and O exhibited a dumbbell‐shaped distribution, while C showed an opposite distribution with silicon and oxygen (Figure 3).

### TEOS Yield obtained from differently treated rice straw

**Figure 4:**
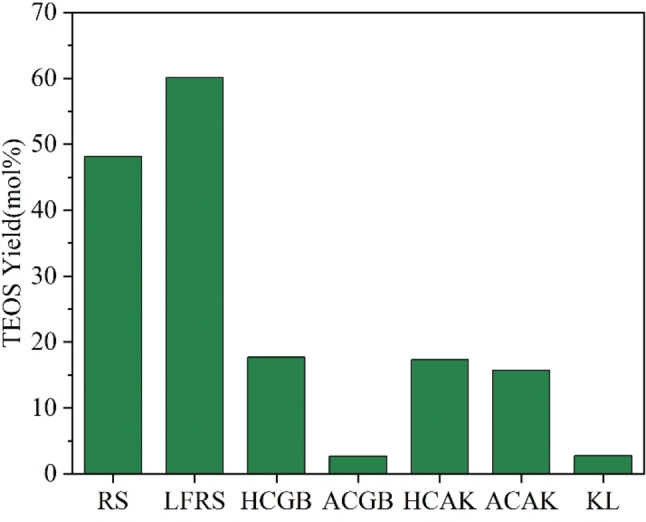
The yield of TEOS obtained from RS and differently treated RS. Reaction conditions: material (100 mg), ethanol (10 mL), 280 °C, 2 MPa N_2_, 2 h. RS, raw rice straw; LFRS, lipids‐free rice straw; HCGB, GB method prepared holocellulose; ACGB, GB method prepared alpha‐cellulose; HCAK, alkali method prepared holocellulose; ACAK, alkali method prepared alpha‐cellulose; KL, Klason‐lignin.

**Figure 5:**
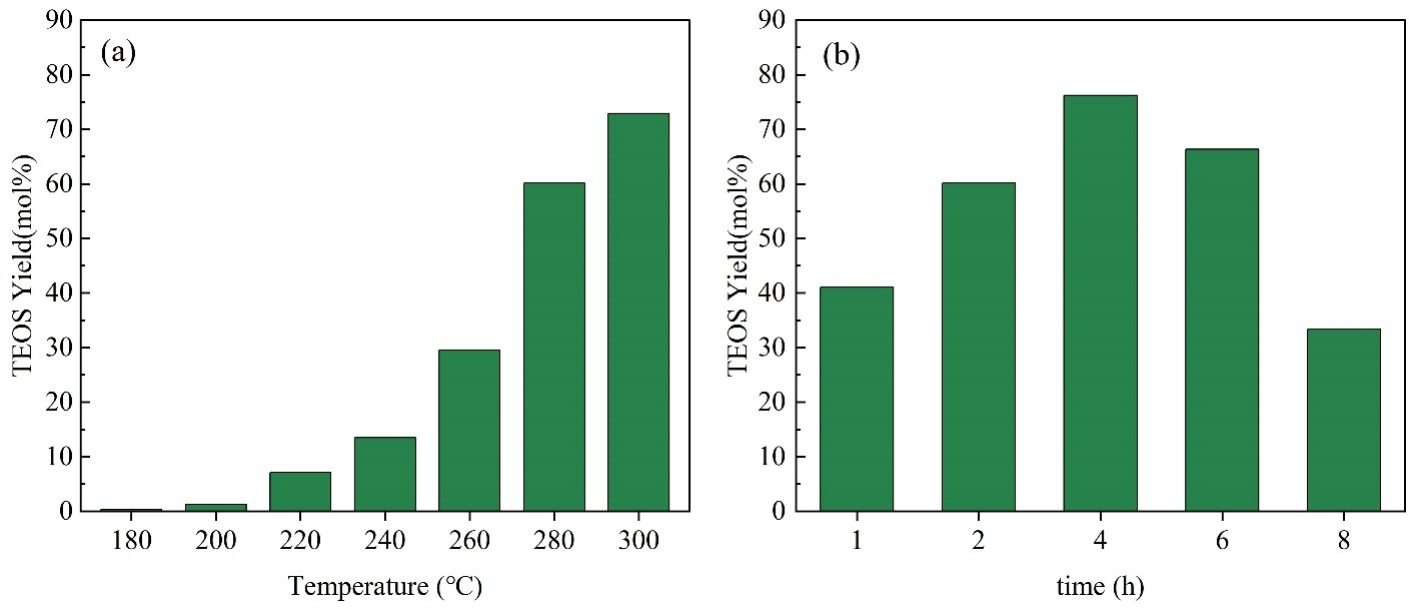
The variation of TEOS yield from LFRS with reaction temperature (a) and time (b). Reaction condition: LFRS (100 mg), ethanol (10 mL), 2 MPa N_2_, 2 h (a), 280 °C (b).

GC‐MS spectra showed that the peak at 7.7 min was due to TEOS (Figure S1), and the ^29^Si NMR spectra also proved the presence of TEOS (Figure S2). In addition to TEOS, several co‐products came from organic components, that is, the phenolics from lignin, the methoxysilane from methoxy group in lignin and Si, and others from cellulose and hemicellulose were also observed, which deserved further study (Figure S3 and S4). The yields of the TEOS from RS and differently treated RS were shown in Figure 4. Untreated RS had a TEOS yield of 48.1 %. After lipids extraction, although 4.4 % Si was lost, Si contained in LFRS was more easily converted to TEOS, and the yield of TEOS increased to 60.1 %. TEOS yields from LFRS at different reaction temperatures and times were shown in Figure 5.

There were nearly no TEOS formed at 180 °C and the yield of TEOS increased with increasing temperature. TEOS yield increased slowly when the temperature increased to 300 °C and the yield was 72.9 %. Although the generation of TEOS has a strong relationship with temperature and high temperature favored the reaction, considering the safety issues, the reaction temperature was set at a maximum of 300 °C. For reaction times, we found that with the increase of time, the yield of TEOS increased. The highest TEOS yield (76.2 %) was obtained in 4 h. Then TEOS yield decreased with further increase of time. This could be caused by the conversion of the generated TEOS to other by‐products. Although, high yield of TEOS was obtained, the separation and purification deserved further study. In our experiment, both rotary evaporator and atmospheric distillation were used to treat the reaction mixture. It was found that even with atmospheric pressure distillation, there was some loss of TEOS.

Biomass consists of lignin, cellulose, and hemicellulose. TEOS production from silicon sources in different components was different. Although the majority of Si in RS remained in HCGB in the treating process, the TEOS yield from HCGB was lower than RS. Whereas the Si remained in HCAK was little, the TEOS yield from ACAK was similar to that from HCGB. The TEOS yield from HCGB and HCAK decreased to 17.7 % and 17.3 %, respectively. The TEOS yield from KL was even lower, although 95.3 % of Si in RS was kept. The above data indicated that the conversion of the Si present in HCGB, HCAK, and KL was not easy.

Moreover, after the removal of hemicellulose, the TEOS yield from ACGB and ACAK decreased to 2.7 % and 15.7 % significantly. Among the differently treated rice straw, Si existed in KL was the most difficult to generate TEOS, followed by those in cellulose (ACGB, ACAK) and holocellulose (HCGB, HCAK). Except for LFRS, the differently treated samples, all have Si loss, and the Si contained in them became difficult to be converted to TEOS. This indicated that the simultaneous conversion of the three components of rice straw facilitated the production of TEOS.

Correlation analysis among various factors were shown in Figure S5. Ash was dominated by silicon, the content of which presented a strongly positive correlation with Si content (r=0.92, p<0.01) (see Figure S5 and Table S2). We found there was no obvious relation between ash content and TEOS yield (r=0.51, p>0.05), and there was also no obvious relation between Si content and TEOS yield (r=0.13, p>0.05). This suggested that the production of TEOS was not related to the amount of Si, but rather to the state of Si existed or other factors.

Si was deposited as an amorphous opal (SiO_2_) in the dumbbell‐shaped silica body of rice straw or bounding with lignin‐polysaccharides in the state of organic silicon which existed in the cell wall during the process of biosilicification.^[36]^ According to the above data, the generation of TEOS may be associated with the state of silicon in the material. Therefore, the relationship between TEOS generation and silicon state was analyzed. SiO_x_ was the predominant surface state of Si for KL (Figure 2g). The low TEOS yield from KL indicated that fully SiO_x_ species were not conducive to TEOS generation. This implied that generating TEOS from SiO_2_ was not possible in this reaction system, though the synthesis of alkoxysilanes from SiO_2_ has been reported, where both strong alkali and dehydrating agents were added in the synthesis process.[^17^, ^39^, ^40^, ^41^] Thus, the reactions that occurred in the present reaction system were different from those in the reported ones. In addition, Si−O−Si species in HCGB, ACGB, HCAK, ACAK, and KL were decreased compared to RS and LFRS, and the TEOS yields were also lower than those from RS and LFRS. It was supposed that the state of Si−O−Si was beneficial to the generation of TEOS. Meanwhile, the correlation between the content of Si−O−Si species and TEOS yield showed strongly positive. (r=0.92, p<0.01) (see Figure S5 and Table S2). This further proved that the state of Si−O−Si was the most crucial to the generation of TEOS. In 1985, researchers showed that there may be a tight layer of extremely fine and highly reactive silica particles that cover the outer surface of the silica body as a ′skin′ using staining methods.^[20]^ In addition, Norihisa Fukaya et al. used rice hull ash to prepare TEOS in the presence of strong alkali, they found the larger the specific surface area of rice hull ash, the higher the TEOS yield.^[18]^ As we know, the specific surface area of the particles is related to the size of the particles, and the smaller the particle, the larger the specific surface area. Meanwhile, the amounts of Si−O−H species present on the silica surface were more. We tried to observe these silicon particles with SEM, but these silica particles were not observed in LFRS due to the covering of lignin on the surface. Fortunately, a small amount of extremely fine silica particles which covered the dumbbell‐shaped silica cells were found in HCGB, where a higher yield of TEOS (17.7 %) than from other separated components was obtained, and it was analyzed by SEM‐EDS (Figure S6). We speculate that the state of Si−O−Si beneficial to the generation of TEOS may be related to these tiny silica particles.

It has been proposed that alcoholysis of silica includes the formation of hypervalent silicon species. According to experimental data, Si−O−Si species might be highly reactive, while, Si−O−H species on the silica surface particles might have high reactivity because H was smaller than Si atoms, thus, Si−O−H species were more likely to form hypervalent silicon species. However, for the state of Si−O−Si and Si−O−H, Si 2p XPS cannot clearly distinguish them.

Another interesting phenomenon observed was that in RS and LFRS, the content of Si−O−Si species was less than the yield of TEOS. This meant that the Si−O−C and SiO_x_ species were also involved in the generation of TEOS. This suggested that the conversion of biomass also affects the conversion of silicon, and the combined effect of the conversion of the three main organic components of biomass will promote the Si−O−C and SiO_x_ species to generate TEOS. This makes it possible to add additional silica to the biomass, and then be converted to TEOS in biomass conversion.

## Conclusions

Silicon in rice straw had different outcomes in different treatment processes. Acid treatment and ethanol extraction resulted in most of the Si being retained in the solid, whereas alkali treatment resulted in significant Si dissolution. The state of Si cannot be fully retained after treatment. For RS and LFRS, Silicon was mainly in the state of Si−O−Si, and with the removal of lignin and hemicellulose, the content of Si−O−Si species decreased. Si−O−C was the main state for silicon in separated cellulose, and for KL, SiO_x_ was the most predominant state of Si. For different states of Si, the Si−O−Si species could be converted to TEOS most easily. Furthermore, lipids‐free rice straw favored TEOS production, giving the highest TEOS yield. Besides, it was found that the combined effect of the conversion of the three components of biomass will promote the Si−O−C and SiO_x_ species to generate TEOS. This makes it possible to add additional silica to the biomass conversion system, being converted into TEOS with the conversion of biomass.

## Experimental Section

### Materials

Rice straw: Rice straw (RS) was obtained from Suqian, Jiangsu Province, China. The rice straw was mechanically ground into powder between 40 and 80 meshes. All reagents were obtained from Chengdu Kelong Chemical Co., Ltd. without further purification.

### Treatment of rice straw

Lipids‐free rice straw (LFRS): First, ground rice straw was treated using a Soxhlet extractor to extract the lipids for 16 h at 95 °C with absolute alcohol solvent. After extraction, the solid fraction was dried at 105 °C for 6 h to obtain LFRS. The liquid fraction was evaporated using a rotary evaporator to obtain lipids. Then, lipids were dried at 40 °C for 6 h in a vacuum oven. The C, H, O, and N in lipids were determined by EA (Euro EA 3000), and the weight percentages of them were 38.1 *wt*. %, 6.3 *wt*. %, 54.5 *wt*. %, and 1.1 *wt*. %, respectively.

Holocellulose and alpha‐cellulose were prepared from LFRS using modified GB method (National Standard method GB/T 2677.10‐1995 and GB/T 744‐1989, respectively.), where ethanol solution was used to extract lipids, instead of using mixed solutions (benzene and ethanol in a 2 : 1 ratio by volume) according to the standard, and the resultant holocellulose and alpha‐cellulose were named HCGB and ACGB.

Holocellulose and alpha‐cellulose were also prepared from LFRS using the alkali method. Firstly, LFRS was mixed in a solution containing 0.7 *wt*. % H_2_O_2_ and 4 *wt*. % NaOH, and heated in a water bath for 4 hours at 85 °C. After the reaction, solids and liquids were separated by centrifugation at 5000 rpm for 5 min. The liquid was decanted, and water was added to wash the solid until the liquid was neutral. The solid was dried in an oven at 105 °C for 6 h. Secondly, the solid was mixed in an acetic acid solution (31 mL/L) containing 8.5 g/L NaClO_2_, and heated in the water bath at 75 °C for 1 hour, then the mixture was centrifuged, washed to neutral, and dried in an oven at 105 °C for 6 h to obtain holocellulose, named HCAK. HCAK was heated in 17.5 *wt*.% NaOH solution in a water bath at 55 °C for 1.5 hours, then the mixture was centrifuged, washed to neutral and dried in an oven at 105 °C for 6 h to obtain alpha‐cellulose, which was named ACAK.

Klason lignin (KL): The KL was obtained from LFRS according to the National Renewable Energy Laboratory (NREL) analytic methods.^[42]^ LFRS (0.24 g) was treated in 72 % H_2_SO_4_ at 30 °C for 120 min, then diluted to 4 % H_2_SO_4_, and autoclaved at 121 °C for 60 min. The hydrolysate was filtered with sand core funnel, and the insoluble residue “Klason lignin” was washed with deionized water to neutral condition. The sand core funnel and Klason lignin were dried in oven at 105 °C for 10 h. After that, KL was obtained.

### Characterization and Experimental Operation

For the analysis of chemical components, the content of lignin, cellulose and hemicellulose were obtained using National Renewable Energy Laboratory (NREL) analytic methods. The sample (0.24 g) was treated in 72 % H_2_SO_4_ at 30 °C for 120 min, then diluted to 4 % H_2_SO_4_, and autoclaved at 121 °C for 60 min, filtered with sand core funnel, which was pre‐dried in an oven at 105 °C until a constant weight obtained. Then, the solid residue was dried in an oven at 105 °C to obtain the weight of acid‐insoluble lignin (AIL), and calcined in a crucible at 625 °C for 2 h to determine the Si content. The acid‐soluble lignin (ASL) in the hydrolysate was analyzed by ultraviolet (UV, U‐4100) spectroscopy, whereas High‐Performance Liquid Chromatography (HPLC, Waters‐e2695) was used to obtain products derived from sugar, and then the content of cellulose and hemicellulose can be quantified with sugar products. ICP‐AES was also used to analyze the content of Si in HCGB, ACGB, HCAK, and ACAK, which cannot be tested by the method above. The biomass was calcined at 625 °C for 2 h in a crucible to get the ash. Then, the ash was dissolved in concentrated hydrochloric acid and heated properly before tests. Fourier transform infrared spectroscopy (FTIR) measurement was carried out using Nicolet Nexus 670 Fourier. The tested range was 4000–400 cm^−1^. and the resolution was 2 cm^−1^. XPS was used to analyze the chemical state of surface species and an AXIS Ultra DLD (KRATOS) spectrometer with a monochromatic Al Kα X‐ray source was used to record the spectra. The samples were tested using an Apreo S (Thermo scientific) combine OXFORF 80 instrument with an acceleration voltage of 20 kV to get the SEM image. All mass spectra were acquired on an AB SCIEX QTOF X500R (AB Sciex Pte. Ltd.) with an electrospray ionization (ESI) probe and operated with spray voltage of 5.5 kV at 500 °C, and accumulation time of 0.1 s. NMR spectra were recorded on a Bruker AVANCE 600 MHz spectrometer. Before NMR measurement, the liquid of the reaction mixture (40 mL) was distilled at 80 °C using a water bath to remove the reaction solvent (ethanol) under atmospheric pressure, and the obtained products were added in 0.6 mL DMSO‐d_6_ to perform NMR.

The reaction was performed in a 50 mL reactor (purchased from Beijing Century Senlong experimental apparatus Co., Ltd), using the biomass samples obtained above. In a typical run, 100 mg biomass sample and 10 mL ethanol solvent were added to the reactor. 2 MPa N_2_ was inflated and exhausted three times and finally filled with 2 MPa N_2_ for reaction. The reaction endured for two hours at 280 °C with stirring at 400 r min^−1^, then, the reactor was cooled to room temperature, and the mixture was filtered. The solution was analyzed qualitatively using GC‐MS (Agilent 6820) and quantitatively using GC equipped with a flame ionization detector (GC‐FID; PERKINELMER Clarus 580). The column of GC and GC‐MS were both DB‐5 capillary columns (30 mm×0.25 mm×0.25 μm). Nitrogen carrier gas flow rate: 1 mL min^−1^, temperature programming: ramp of 5 °C min^−1^ from 50 to 250 °C and maintaining for 10 min. The injector and detector temperatures were 280 and 330 °C. Benzaldehyde was used as an internal standard, and the correction factor was 0.5433. The yield of tetraethyl orthosilicate (TEOS) was calculated by Equation (1). 1

(1) YieldofTEOS(mol%)=(molofTEOS)/(molofSiinbiomass)×100%

## Supporting Information

The Supporting Information contains a table of literature research on the production of alkoxysilanes. GC‐MS spectra of reaction liquid of lipids‐free rice straw. Correlation analysis among various factors and TEOS yield. SEM‐EDS analysis of HCGB. ^1^H, ^13^C, and ^29^Si NMR‐600 M of the reaction liquids from lipids‐free rice straw. ESI‐MS of the reaction products from lipids‐free rice straw.

## Conflict of interest

The authors declare no conflict of interest.

1

## Supporting information

As a service to our authors and readers, this journal provides supporting information supplied by the authors. Such materials are peer reviewed and may be re‐organized for online delivery, but are not copy‐edited or typeset. Technical support issues arising from supporting information (other than missing files) should be addressed to the authors.

Supporting Information

## Data Availability

The data that support the findings of this study are available from the corresponding author upon reasonable request.
