# Supplementary material for: Influence of Different Treatments on the Structure and Conversion of Silicon Species in Rice Straw to Tetraethyl Orthosilicate (TEOS)
Source: ChemistryOpen. 2023 Aug 7;12(8):e202300111. doi: 10.1002/open.202300111 (PMC10407258; doi:10.1002/open.202300111)
Supplement: Supplementary file 1 — Supporting Information [file OPEN-12-e202300111-s001.pdf]

ChemistryOpen

Supporting Information

Influence of Different Treatments on the Structure and Conversion of Silicon Species in Rice Straw to Tetraethyl Orthosilicate (TEOS)

Qianxin Sun, Shanshan Feng, Guiying Li, Yue Qi, and Changwei Hu*

Supplementary Table 1 The production of alkoxysilanes

Raw materials	Reaction steps	Catalyst	Reagents involved	Condition	Product	Yield	literature
SiO_2(via $SiCl_4$)	4	Cu/Sn	C, HCl, EtOH	>1700°C	Tetraethyl Orthosilicate (TEOS)	[a]	43
Si	2	CuCl, Cu	MeOH	380 °C, 1 h	Tetramethoxysilane	0.4 %	44
Si	2	CuCl, $CuCl_2$, CuO, Cu	Isopropyl titanate, EtOH	350 °C, 7 h; 180 °C, 7 h	TEOS	97 %	45
γ-Ca_2SiO_4	2	-	HCl, Toluene, EtOH	40 °C	TEOS	33 %	46
Ca_3SiO_4O	2	-	HCl, Toluene, EtOH	40 °C	TEOS	42 %	46
SiO_2	1	KOH (5-10%)	Dimethyl carbonate	320 °C, 1 h	Tetramethoxysilane	20 %	47
SiO_2	2	NaOH, etc (10 mol%)	Ethylene glycol, EtOH	200 °C	TEOS	60 %	17
SiO_2	1	KOH (10 mol%)	CO_2, acetal, MeOH	260 °C, 24 h	Tetramethoxysilane	59 %	48
SiO_2	1	KOH (10 mol%)	Molecular Sieves, EtOH	260 °C, 6 h	TEOS	70 %	18
SiO_2	1	KOH	3A Molecular Sieves, EtOH	270 °C	Tetramethoxysilane	36.2 %	40
**Rice straw**	**1**	**-**	**EtOH**	280 °C, 4 h	**TEOS**	**76.2 %**	**This work**

[a] current industrial process, value not available; [-] nothing added

(a)

(b)

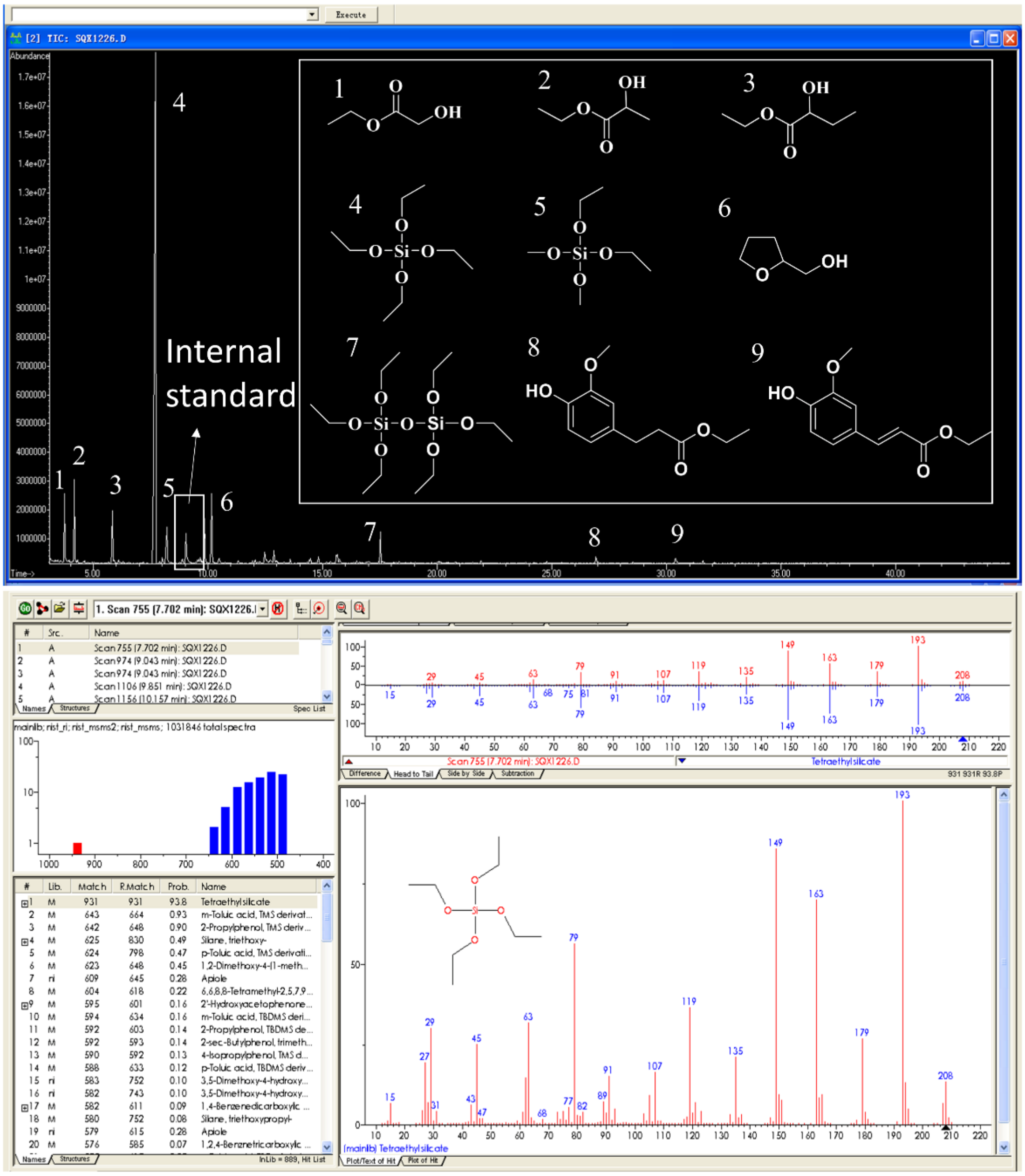

Supplementary Figure 1 a) GC-MS spectra of reaction liquid of lipids-free rice straw at 280°C and 2 h. b) The GC-MS spectrum data for TEOS. Benzaldehyde was used as an internal standard, and the correction factor was 0.5433.

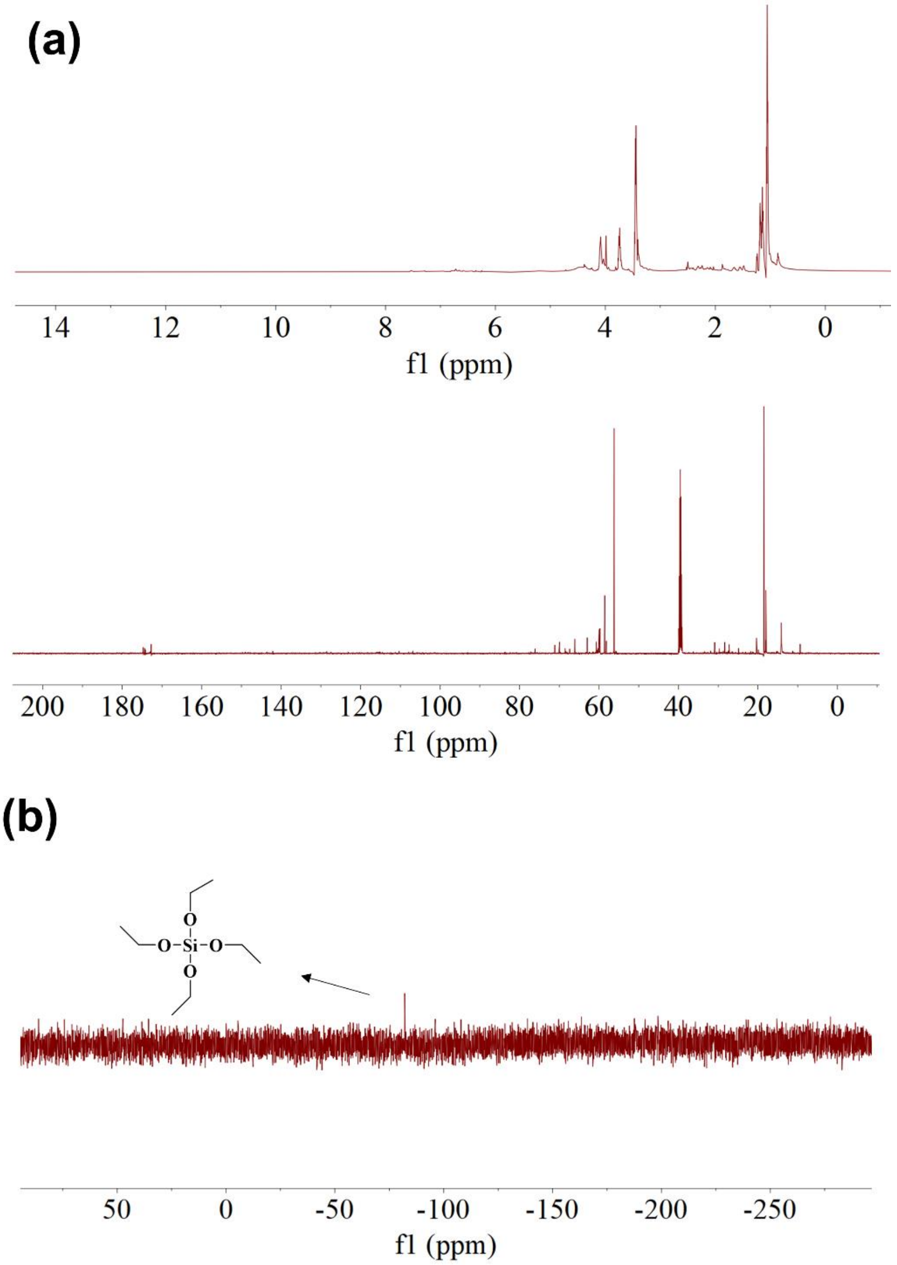

Supplementary Figure 2 a) ^{1}H and ^{13}C NMR-600M of the reaction liquids from lipids-free rice straw b) ^{29}Si NMR-600M spectra of the reaction liquids from rice straw. Reaction condition: 280 °C, 2 h, 2 MPa N_2.

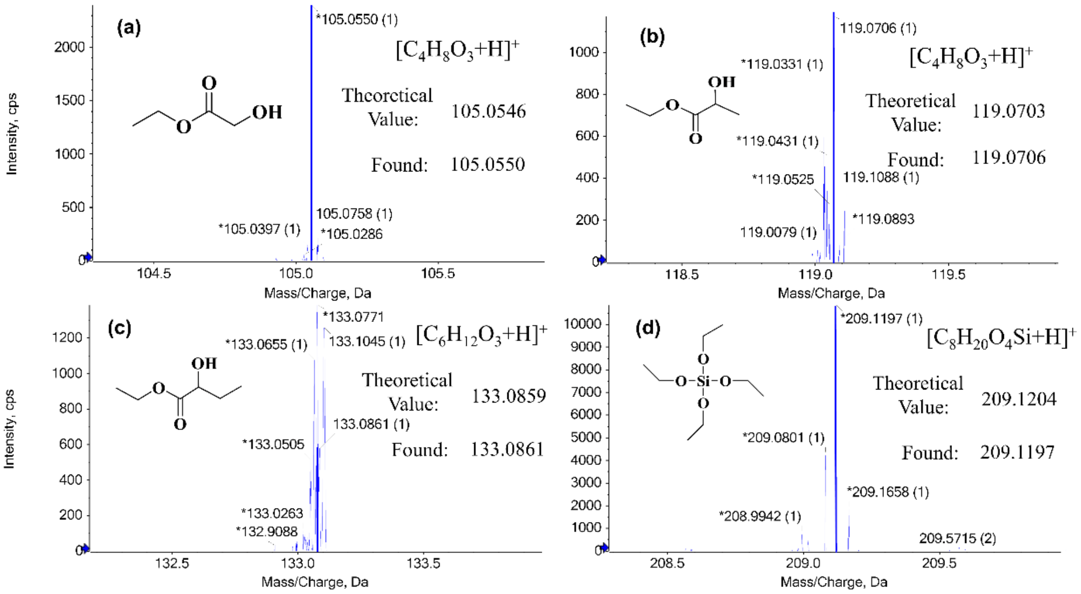

Supplementary Figure 3 ESI-MS of the reaction products from lipids-free rice straw: a) Ethyl glycolate, b) Ethyl lactate, c) Ethyl 2-Hydroxybutyrate, d) TEOS. Reaction condition: 280 °C, 2 h, 2 MPa N_2.

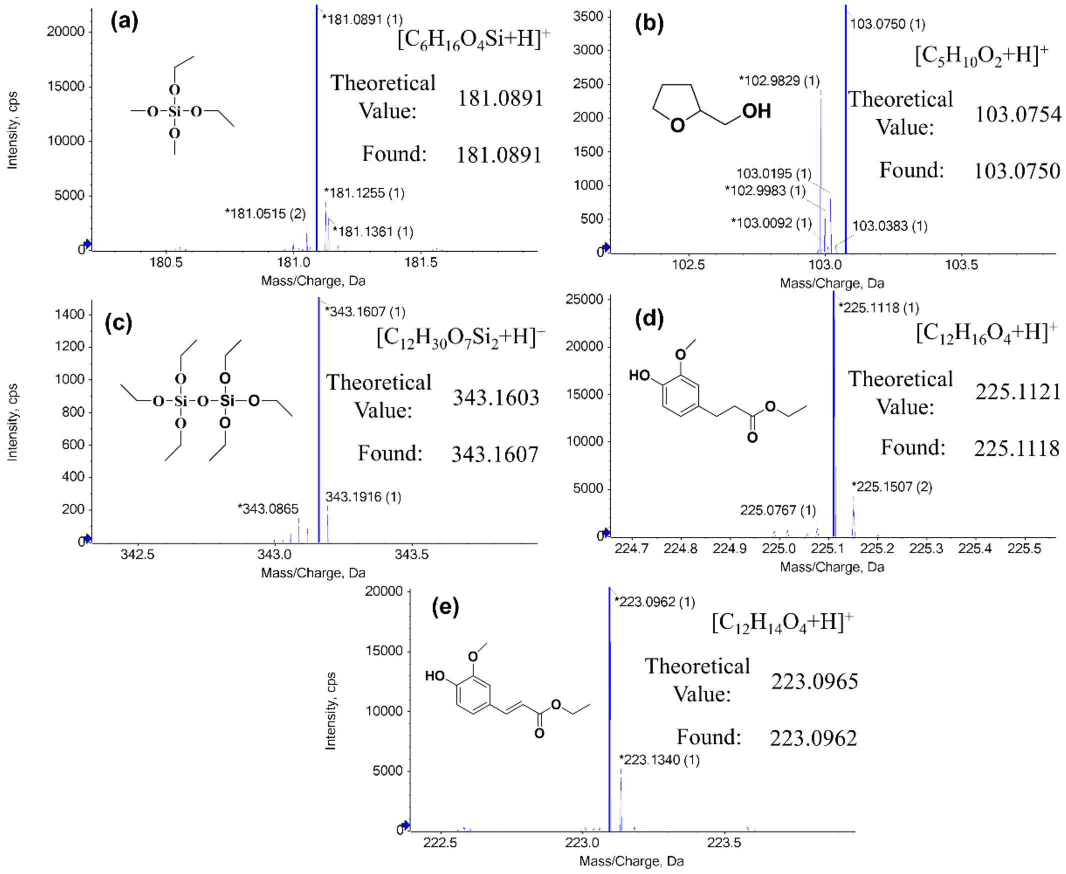

Supplementary Figure 4 ESI-MS of the reaction products from lipids-free rice straw: a) Diethoxy dimethoxy silicate, b) (tetrahydrofuran-2-yl) methanol, c) hexaethyl disilicate, d) ethyl 3-(4-hydroxy-3-methoxyphenyl) propanoate, e) ethyl (E)-3-(4-hydroxy-3-methoxyphenyl) acrylate. Reaction condition: 280 °C, 2 h, 2 MPa N_2.

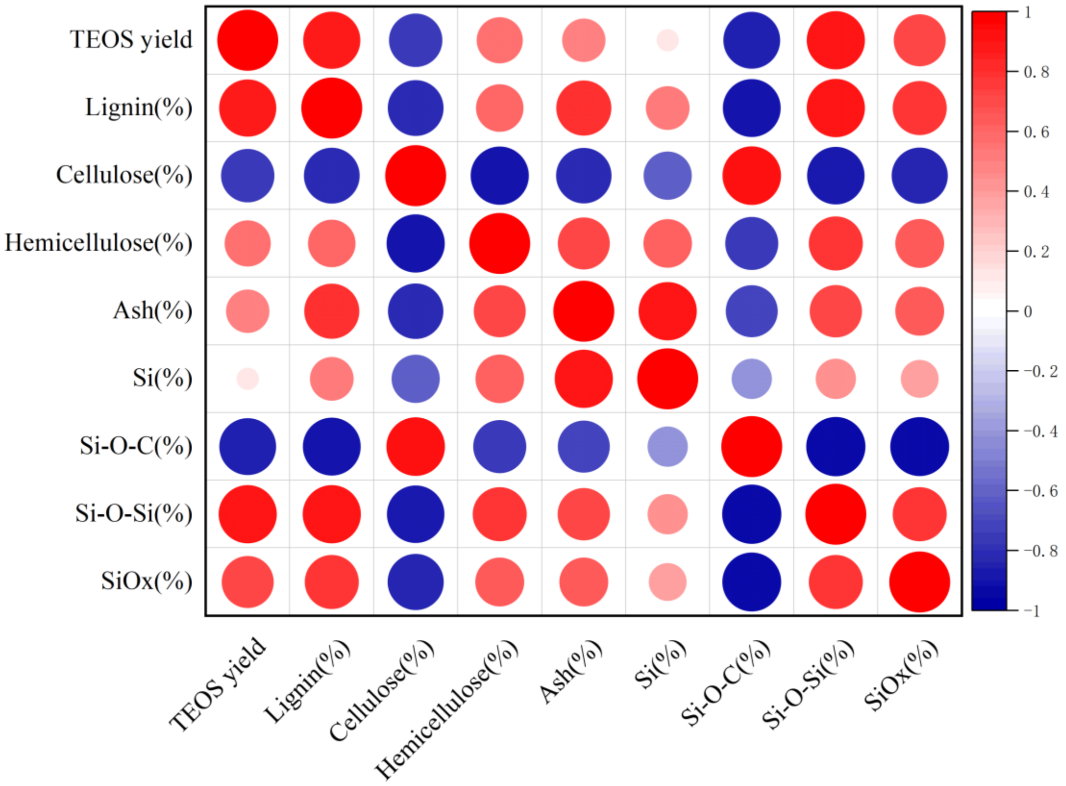

Supplementary Figure 5 Metrix of correlation coefficients among various factors and TEOS yield. Larger redder (bluer) plots display stronger positive (negative) correlations.

Supplementary Table 2 Correlation analysis among all factors

		TEOS yield	Lignin (%)	Cellulose (%)	Hemicellulose (%)	Ash (%)	Si (%)	Si-O-C (%)	Si-O-Si (%)	SiOx (%)
TEOS yield	Pearson correlation	1	0.88	–0.77	0.57	0.51	0.13	–0.88	0.92	0.73
	Significance	-	0.020	0.076	0.230	0.300	0.800	0.022	0.010	0.097
	N	16	16	16	16	16	16	16	16	16
Lignin (%)	Pearson correlation	0.88	1	–0.84	0.61	0.82	0.52	–0.91	0.92	0.79
	Significance	0.02	-	0.038	0.200	0.046	0.290	0.013	0.010	0.061
	N	16	16	16	16	16	16	16	16	16
Cellulose (%)	Pearson correlation	–0.77	–0.84	1	–0.91	–0.83	–0.63	0.92	–0.9	–0.85
	Significance	0.076	0.038	-	0.011	0.042	0.180	0.008	0.016	0.031
	N	16	16	16	16	16	16	16	16	16
Hemicellulose (%)	Pearson correlation	0.57	0.61	–0.91	1	0.73	0.63	–0.76	0.79	0.64
	Significance	0.230	0.200	0.011	-	0.100	0.180	0.078	0.060	0.17
	N	16	16	16	16	16	16	16	16	16
Ash (%)	Pearson correlation	0.51	0.82	–0.83	0.73	1	0.92	–0.73	0.74	0.65
	Significance	0.300	0.046	0.042	0.100	-	0.010	0.097	0.094	0.160

	N	16	16	16	16	16	16	16	16	16
	Pearson correlation	0.13	0.52	–0.63	0.63	0.92	1	-0.44	0.44	0.39
Si (%)	Significance	0.800	0.290	0.180	0.180	0.01	-	0.380	0.380	0.450
	N	16	16	16	16	16	16	16	16	16
	Pearson correlation	–0.88	–0.91	0.92	–0.76	–0.73	–0.44	1	–0.95	–0.94
Si-O-C (%)	Significance	0.022	0.013	0.008	0.078	0.097	0.380	-	0.004	0.005
	N	16	16	16	16	16	16	16	16	16
	Pearson correlation	0.92	0.92	–0.90	0.79	0.74	0.44	–0.95	1	0.79
Si-O-Si (%)	Significance	0.010	0.010	0.016	0.060	0.094	0.380	0.004	-	0.062
	N	16	16	16	16	16	16	16	16	16
	Pearson correlation	0.73	0.79	–0.85	0.64	0.65	0.39	–0.94	0.79	1
SiOx (%)	Significance	0.097	0.061	0.031	0.170	0.160	0.450	0.005	0.062	-
	N	16	16	16	16	16	16	16	16	16

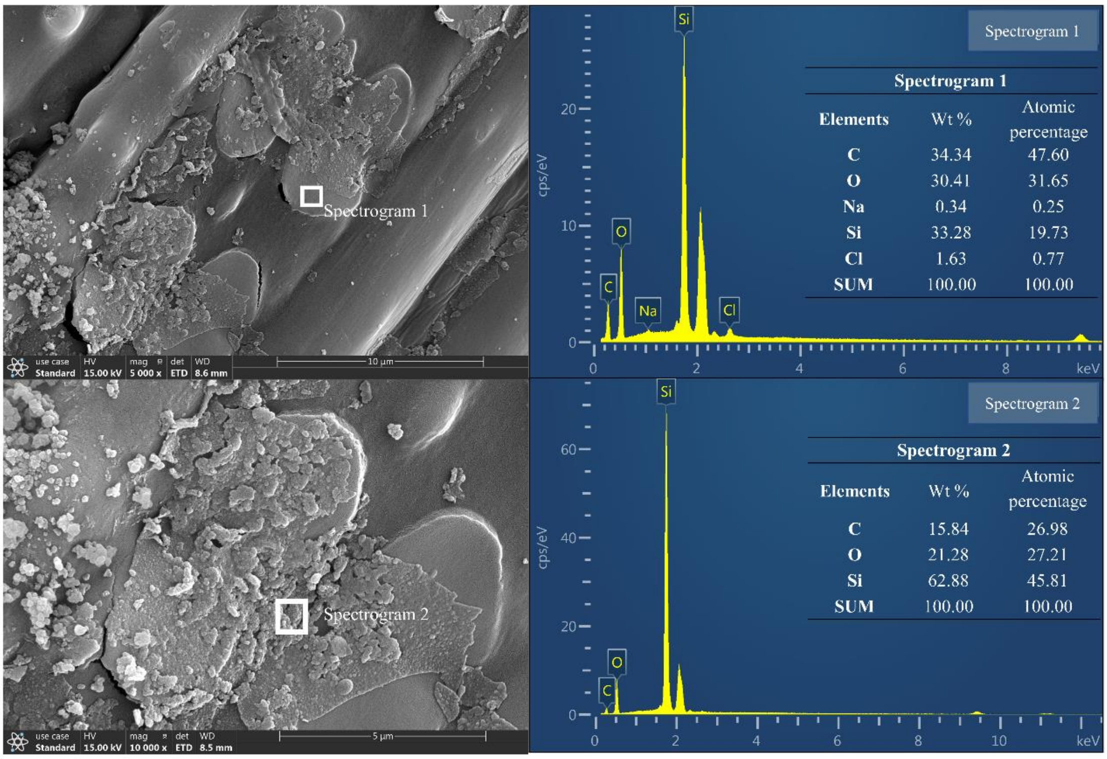

Supplementary Figure 6 SEM-EDS analysis of HCGB.
